# Translocation of the neonicotinoid seed treatment clothianidin in maize

**DOI:** 10.1371/journal.pone.0173836

**Published:** 2017-03-10

**Authors:** Adam Alford, Christian H. Krupke

**Affiliations:** Department of Entomology, Purdue University, West Lafayette, Indiana, United States of America; Louisiana State University, UNITED STATES

## Abstract

Neonicotinoid seed treatments, typically clothianidin or thiamethoxam, are routinely applied to >80% of maize (corn) seed grown in North America where they are marketed as a targeted pesticide delivery system. Despite this widespread use, the amount of compound translocated into plant tissue from the initial seed treatment to provide protection has not been reported. Our two year field study compared concentrations of clothianidin seed treatments in maize to that of maize without neonicotinoid seed treatments and found neonicotinoids present in root tissues up to 34 days post planting. Plant-bound clothianidin concentrations followed an exponential decay pattern with initially high values followed by a rapid decrease within the first ~20 days post planting. A maximum of 1.34% of the initial seed treatment was successfully recovered from plant tissues in both study years and a maximum of 0.26% was recovered from root tissue. Our findings show neonicotinoid seed treatments may provide protection from some early season secondary maize pests. However, the proportion of the neonicotinoid seed treatment clothianidin translocated into plant tissues throughout the growing season is low overall and this observation may provide a mechanism to explain reports of inconsistent efficacy of this pest management approach and increasing detections of environmental neonicotinoids.

## Introduction

The neonicotinoids are a relatively new group of systemic insecticides. The first commercially available compound, imidacloprid (Bayer CropScience), was available in the early 1990s, with other compounds following in the 2000s. They have since become the most widely used insecticide class worldwide [1,2]. Their rapid and widespread adoption has been attributed to low mammalian toxicity, systemic and translaminar properties, lack of resistance upon market entry, increasing restrictions and regulations on older pesticide groups, and potential for a wide variety of application methods [3]. Neonicotinoids are most frequently used as seed treatments (ST), comprising 80% of the ST market worldwide in 2008 [1]. Maize (corn), along with the other three major US field crops (soybean, wheat, and cotton) by area planted [4], all have neonicotinoid seed treatment (NST) registrations using the active ingredients (AI) imidacloprid, clothianidin (CLO) (Bayer CropScience), and thiamethoxam (Syngenta Crop Protection) [5].

The neonicotinoids are used in US maize production solely as STs, with >80% of maize planted annually being treated with either CLO or thiamethoxam at application rates of 0.25–1.25 mg/kernel prior to sale to the grower [5,6]. While Bt hybrids largely control damage from the western corn rootworm (*Diabrotica virgifera virgifera* LeConte) [7,8], the primary maize pest of Indiana [9] (recent resistance notwithstanding [10]), the 1.25 mg/kernel rate is also labeled to control the larval stage of this pest [11,12]. NSTs are also labeled to control a range of other secondary, early season root and seed pests including wireworms [13], seedcorn maggots [14], and white grubs [15]. White grubs preferentially attack the root tissue of young seedlings [16] and wireworms occasionally burrow into the stems of young seedlings [17] however, both will readily feed on the germinating seed [18], the primary site of seedcorn maggot attack [14]. While seedcorn maggot injury pressure can be reliably predicted based upon incorporation of a green cover crop into the soil [19], economic infestations of these secondary pests are typically sporadic and difficult to predict [17]. This has in part, led to the widespread adoption of NSTs as a prophylactic measure of minimizing pest damage risk.

Both CLO and thiamethoxam are hydrolytically stable with relatively high respective solubilities of 0.327 g/L at 20°C and 4.1 g/L at 25°C; these solubilities confer systemic properties [20]. Despite widespread use in maize systems, little has been published on the translocation efficiency of the NST delivery method in maize, or the distribution and concentration of these compounds throughout the plant once material is translocated [21]. A potted plant study [22] found thiamethoxam shoot concentrations in maize to be 0.62 μg thiamethoxam/g 21 days post plant (DPP) with a gradual concentration decrease to 0.13 μg thiamethoxam/g at 36 DPP from an initial 0.1 mg thiamethoxam per kernel ST. Root tissue thiamethoxam was not quantified nor ST efficacy, viewed in terms of the percentage of initial ST translocated to plant tissue. Furthermore it is unknown what concentrations would provide a pest management benefit. To inform the debate surrounding the costs and benefits of this management approach, these data represent key parameters in defining a “pest management window” where these products could be expected to provide crop protection, as well as informing environmental fate studies once the AI is liberated from the seed.

Previously published greenhouse studies examining imidacloprid ST in maize report 20% of the AI being translocated into the plant with the remaining 80% assumed to enter the soil matrix and/or leach away from the plant and its root zone [23]. Once applied and within soil, the time required to dissipate 50% of the applied AI (DT_50_) is highly variable within and between neonicotinoid compounds. The DT_50_ of imidacloprid, thiamethoxam, and CLO, the three compounds used in NSTs [3], are 40 [24] to 270 [25], 7.1–92.3 [26], and 277–1386 days [27] respectively under field conditions. There is no published work explaining the high variability in published DT_50_ values [21].

Given their soil persistence and repeated use, concern exists over the potential of neonicotinoids in environmental loading and water contamination via leaching and field runoff [27]. While not the only metric used to assess leaching risk, the Groundwater Ubiquity Score (GUS) [28] relates the compound’s soil organic carbon-water partitioning coefficient (K_oc_), and DT_50_ and assigns high, medium, and low leaching potentials to respective GUS values of >2.8, 1.8–2.8, and <1.8. Both CLO and thiamethoxam have GUS values of 5.43–6.98 and 1.84–4.25 each, based upon respective K_oc_ values of 60 and 68.4 [29,30] and DT_50_ values listed above.

Increasing detections of neonicotinoids in a range of surface and ground waters have been reported. A few of these studies [31–34] suggest water contamination as the direct result of runoff or leaching and in multiple instances, concentrations exceeded either chronic [31–33,35,36] or acute [36] toxicity benchmarks for freshwater invertebrates [37]. Detection of neonicotinoids in non-target vegetation has also been attributed to lateral subsurface movement in the USA [38], the UK [39], and implicated as a pathway for neonicotinoid contamination of organic fields [40]. Although little has been published quantifying the ultimate impact of these compounds in the environment, correlative studies have indicated that these compounds may be causal agents of long-term macroinvertebrate decline in surface water [41], (although this hypothesis has been disputed as not accounting for the presence of other insecticides [42]) and of insectivorous bird populations [43].

The numerous examples of environmental detections of neonicotinoids coupled with a variable soil half-life highlight the potential of these compounds to accumulate in the environment. However, the mechanism is unclear. A key unknown in untangling these mechanisms is the amount of material that enters the target (i.e. crop plants). It is the purpose of this paper to quantify these levels in space (various plant regions) and time (across the early growing season) in order to: 1) define the pest management window afforded by these compounds and 2) determine one component of the environmental fate of the NST. The work described here provides baseline information on the translocation of the major NST of maize, CLO, into various regions of the growing plant, using field-collected plants beginning at seed sowing and continuing through the growing season.

## Methods

### 2014 & 2015 field site and experimental design

Planting of hybrids Dekalb 6179 (2014) and Spectrum 6241 (2015) took place on May 5th at the Throckmorton Purdue Agricultural Center (40°18'00.7"N 86°43'37.0"W). The 5-year precipitation average in May (mean ± sd) for this site is 67.2 ± 27.96 mm and the soil is characterized as loam with a 43.6/38.4/18 sand/silt/clay ratio. Four treatment levels were evaluated: untreated seed (“Naked”), in which no ST was applied, a fungicide only ST (“Fungicide”), a low rate applied at 0.25 mg CLO/kernel (“Low”), and a high rate of 1.25 mg CLO/kernel (“High”). The “Fungicide”, “Low” and “High” treatments were also treated with the fungicides metalaxyl, trifloxystrobin, and ipconazole at respective rates of 0.92, 4.79, and 2.4 g/100 kg of seed. Each treatment level was replicated four times in a randomized complete block design with treatment plots measuring 3.05 x 36.58 m in 2014 and 3.05 x 33.53 m in 2015. The previous crop in both years was a “trap crop” of late planted maize to maximize western corn rootworm egg deposition. The late season “trap crop” was comprised of corn hybrids expressing Bt genes targeting lepidopteran pests and treated with the “Low” CLO rate. Given the instances of subsurface flow [38–40], and proximity of untreated plots to treated plots (plots ~3m wide), CLO contamination of untreated plots was expected and inevitable–a truly neonicotinoid-free field is not achievable in the maize and soybean production areas of North America [5]. Attempts were made to minimize contamination by only collecting samples from the 2 central rows of each plot.

### 2014 & 2015 sampling, root ratings, stand, yield

Sampling consisted of removing ten randomly selected maize plants intact from each plot and storing them at -20˚C for later processing. Five of these ten samples were processed with a modified QuECHeRs protocol [44] (details in S1 Text) with the remaining five serving as reserve samples. At 21 days post planting (DPP), plant tissue CLO concentrations were expected to approach zero based upon preliminary data gathered in 2013, so sampling was reduced to five plants per treatment plot every other week with three of the five samples being processed and analyzed. Sampling was concluded at 61 DPP in both years as CLO concentrations in treated plants were expected to approach the CLO concentrations of untreated plants by this time based upon preliminary data. Increases of in-plant CLO concentrations were not expected either. Stand counts were conducted at the V2/V3 stage on June 3^rd^ and 8^th^ in 2014 and 2015 respectively to assess seed germination. Root damage by the western corn rootworm was scored [45] on July 18th and the 23^rd^ in 2014 and 2015 respectively. An average treatment plot root rating was calculated from four roots in 2014 and five roots in 2015. Maize was harvested and yield calculated on October 10^th^ in 2014 and October 15^th^ in 2015 after adjusting maize moisture to 15.5% [46].

### Calculation of economic damage

The minimum node-injury required to cause economic damage was calculated for both sampling years using the Oleson et al. method [45]. Calculations included a range of insect control costs ($17.5-$55/ha), assumed a moderate level of environmental stress (21.7 heat stress degree-days) and used an average of $14.96/100 kg for market value given the similarity of Indiana maize marketing values between 2014 and 2015 ($14.76 and $15.15/100 kg respectively) [47].

### Sample preparation for chemical analysis

**Fig 1:**
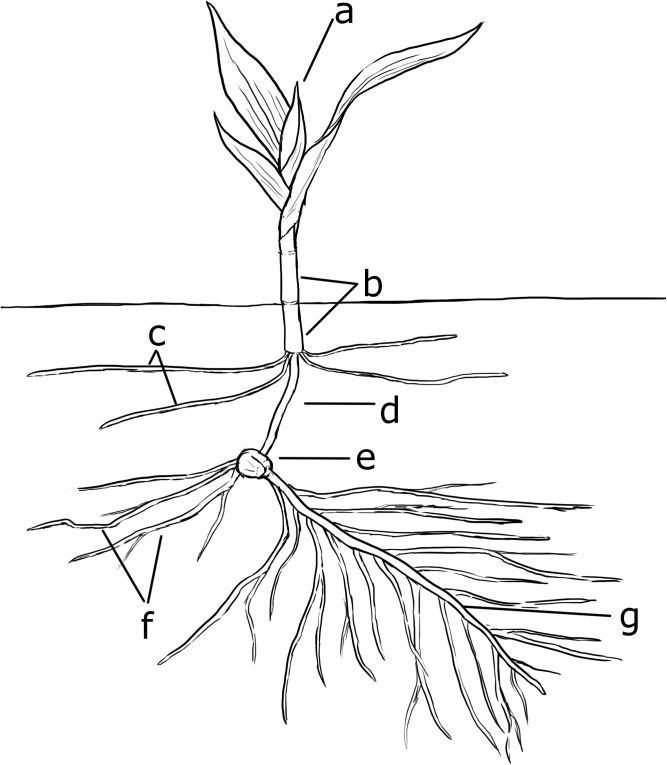
**Diagram of a maize seedling at the soil surface interface, showing (a) Stem apex, (b) coleoptile, (c) nodal roots, (d) mesocotyl, (e) seed, (f) seminal roots, and (g) radicle roots.** For homogenization purposes, the shoot region was classified as sections (a-d), the seed region as (e), and the root region as (f) and (g).

A modified QuECHeRs protocol [44] (details in S1 Text) was used to prepare samples for chemical analysis in both years. Individual samples were split into root, seed, and shoot regions in both years (Fig 1) after residual soil was removed from plant tissue by running the sample underneath a gently running faucet. For samples weighing <1 g per plant region, the root region was considered the radicle and seminal roots, while the shoot region was defined as all plant tissue from the base of the mesocotyl to the stem apex. For samples weighing >1 g per plant region (samples collected after 20 and 16 DPP in 2014 and 2015, respectively), subsections of the stem apex, the area of newest growth, and of a randomly selected root were used for homogenization and further analysis of shoot and root regions, respectively. No more than 1 g of plant tissue was used per tissue region due to space limitations of homogenization tubes. Root and shoot regions for a given sample were scored as “complete” (>80% present) or “incomplete” (<80% present) prior to homogenization. An average % AI translocated for each plant region (root and shoot) was calculated from these data. If both the corresponding root and shoot region of a given plant was scored as “complete”, the plant was scored as a “total sample” and the respective concentrations for both regions were combined to calculate an average overall % AI translocation per plant.

### Determination of pest activity period

The active period of western corn rootworm, seedcorn maggot, wireworm, and white grubs was estimated in both years to compare it to the NST protection window. For western corn rootworm, this was accomplished by checking maize roots daily in a nearby field until observation of neonate western corn rootworm larvae. The seedcorn maggot, wireworm, and white grubs were not directly monitored as they were not present in our study field, and economic infestations are typically sporadic and unpredictable [17]. Egg hatch for seedcorn maggot was estimated with a degree day model [48] used in conjunction with atmospheric data [49]. Adult seedcorn maggot emergence and subsequent oviposition occurs in early May for central Illinois [16], so calculations assumed adult emergence occurred on May 1st for Indiana given the similarity in latitudes. Degree-day models were inappropriate to define an active period for wireworm and white grubs due to their multispecies status so peer-reviewed and extension literature was searched instead [50].

### Statistical analyses

#### Effects of NST on yield, stand count, and root ratings

Yield, stand count, and root ratings were analyzed using SAS PROC MIXED [51] in both years. Treatment and block was included as fixed effects in all six models. A Tukey (HSD) post-hoc analysis was used to separate which treatment means were significantly different from each other (*α* < 0.05) following a significant test result (*P* < 0.05) [52].

#### Determination of protection window

Two different approaches were used to estimate the pest management window. The first approach fit a first order decay function [53] using a Levenberg-Marquardt nonlinear least-squares algorithm with the package minpack.lm in the R statistical language [54,55] to translocation data for each plant region (root, seed, and shoot) as a function of DPP. Decay curves were visually examined to estimate at what DPP the rate of change decreased to where concentration appeared to “flatten out”. Protection was considered lost at this point.

The second, more conservative approach, analyzed translocation data using a multivariate approach to repeated measures with SAS PROC GLM [51]. Prior to analysis, data were natural log transformed to conform to normality assumptions and confirmed with visual inspection of residuals. Separate models were used for each plant region (root, seed, shoot) in both sampling years (2014, 2015). Fixed main effects included treatment, block, sampling date, and a multivariate treatment*sampling date interaction effect as predictors of CLO concentration. Univariate results of the repeated measures ANOVA were analyzed in concert with visual inspection of decay functions to inform designation of appropriate linear contrasts (*α* = 0.05) for determining when the residue curves converged in time. When CLO concentration in treated plants was similar to the untreated controls, any protection afforded by the NST was considered expired. Two sets of contrasts were made: “Naked”+“Fungicide” vs “Low” and “Naked”+“Fungicide” vs “High”.

## Results

### Sampling and effects of NST on yield, stand count, and root ratings

In 2014, sampling and AI extraction was carried out at 6, 8, 10, 13, 15, 17, 20, and 34 DPP and at 5, 7, 9, 12, 14, 16, 19, 47, and 61 DPP in 2015. Freezer failure resulted in no AI extraction past 34 DPP in 2014 samples and resulted in the loss of 33 DPP samples in 2015. The seed region was only recovered up to 20 and 19 DPP in 2014 and 2015 respectively. The limit of CLO detection was determined to be 0.1ng/g.

In 2014, neither treatment or block had a significant effect on root ratings (F_3,9_ = 0.98, *P* = 0.4431; F_3,9_ = 0.83, *P* = 0.5097). For yield, only block had a significant impact (F_3,9_ = 13.65, *P* = 0.001) whereas treatment did not (F_3,9_ = 1.06, *P* = 0.4131). Neither variable had a significant effect (Treatment: F_3,9_ = 0.50, *P* = 0.6937; Block: F_3,9_ = 2.73, *P* = 0.1063) on stand count (Table 1).

**Table 1:** Means of yield, plants per hectare and root ratings for both 2014 and 2015 field season. Within a given column and year, means followed by the same letter denote statistical similarity as determined by Tukey HSD^a^ comparisons.

2014	Yield±SE (kg/ha)	n^b^	Plants per Hectare±SE	n^b^	Root Ratings ±SE	n^b^
Naked	13216±547 a	4	13331±2305 a	4	0.006±0.004 a	4
Fungicide	13741±618 a	4	12737±289 a	4	0.031±0.012 a	4
Low	13997±523 a	4	14610±1302 a	4	0.019±0.008 a	4
High	13743±859 a	4	12935±1037 a	4	0.028±0.017 a	4
2015						
Naked	13159±552 a	4	13398±615 a	4	0.193±0.059 a	4
Fungicide	12072±890 a	3^c^	13398±260 a	4	0.456±0.259 a	4
Low	13423±501 a	3^c^	12208±440 a	4	0.378±0.166 a	4
High	13266±184 a	4	13750±239 a	4	0.128±0.006 a	4

^a^*P* < 0.05.

^b^Number of observations used in calculating mean and SE in previous column.

^c^Data from two plots were lost due to combine malfunction.

In 2015, neither treatment (F_3,9_ = 1.57, *P* = 0.2639) or block (F_3,9_ = 3.44, *P* = 0.0654) had a significant effect on root ratings, stand (Treatment: F_3,9_ = 2.57, *P* = 0.1193; Block: F_3,9_ = 0.93, *P* = 0.4646), or yield (Treatment: F_3,7_ = 0.83, *P* = 0.5192; Block: F_3,7_ = 1.78, *P* = 0.2392) (Table 1).

### Calculation of economic damage

**Fig 2:**
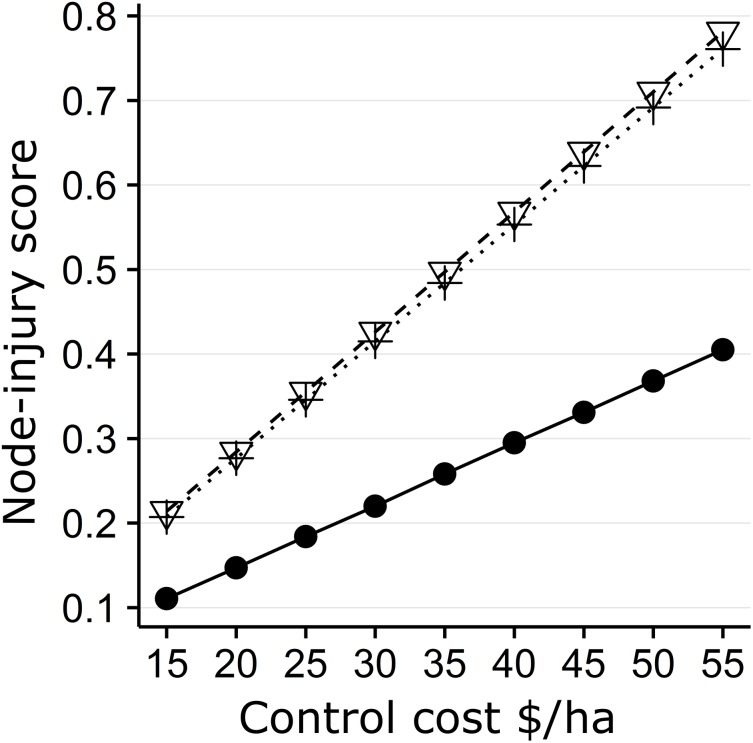
The minimum node-injury score required to cause economic damage calculated according to the Oleson et al. (2005) moderate environmental stress model. 2014 and 2015 are represented as an inverted triangle with dashed lines and crosses with dotted lines respectively. 2012 price data is included to show how recent (five year) high commodity values affect economic thresholds and is represented as a filled circle with solid lines.

The minimum node-injury required to cause economic damage in both sampling years ranged from 0.245–0.771 with a respective control cost of $17.5-55/ha. Economic injury levels were only reached in 2015 in the “Low” and “Fungicide” treatment plots with a control cost <$27/ha and <$32.5/ha respectively (Table 1 and Fig 2).

### Translocation efficacy

**Fig 3:**
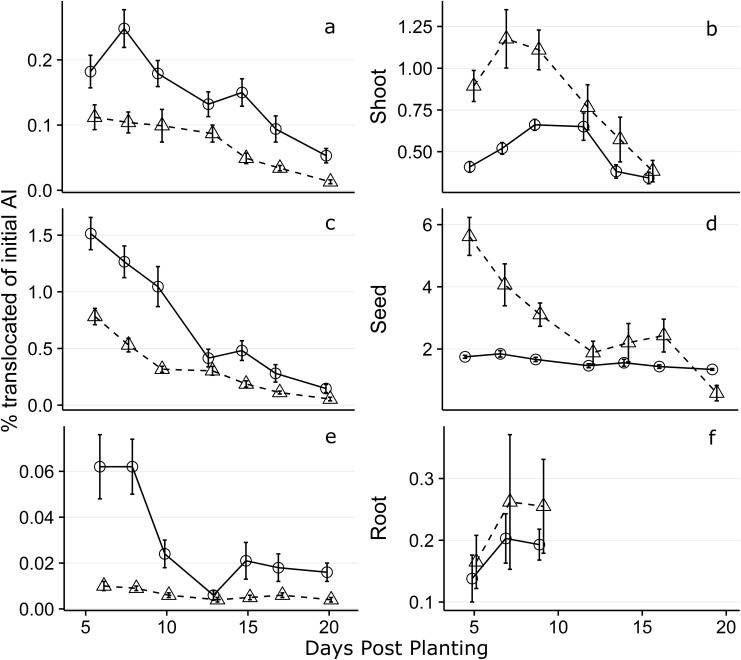
Mean percentage of initial clothianidin application translocated to root, seed, and shoot tissues. The Low and High treatment rates are represented by an open triangle with dashed lines and an open circle with a solid line respectively. Only plants with >80% of root and >80% of shoot tissue were used in calculation of the % of initial AI translocated. The 2014 data represented by graphs (a), (c), and (e), and 2015 data by graphs (b), (d), and (f). The first 20 days post planting (DPP) are shown.

For all plant regions, a greater proportion of the initial CLO seed treatment was successfully translocated in 2015 plants (Fig 3). A maximum of 0.26 and 1.18% of the initial “Low” treatment rate was translocated to respective root and shoot tissues in 2015 whereas a maximum translocation of 0.20 and 0.65% was recovered in the root and shoot region with the “High” treatment. In all instances, less than 1.5% of the initial ST was translocated to the root and shoot region in “total sample” homogenizations. The “High” treatment rate experienced a greater proportion of the initial ST translocation in 2014 but not in 2015, where the “Low” application rate resulted in greater overall translocation.

### Determination of pest activity period

**Fig 4:**
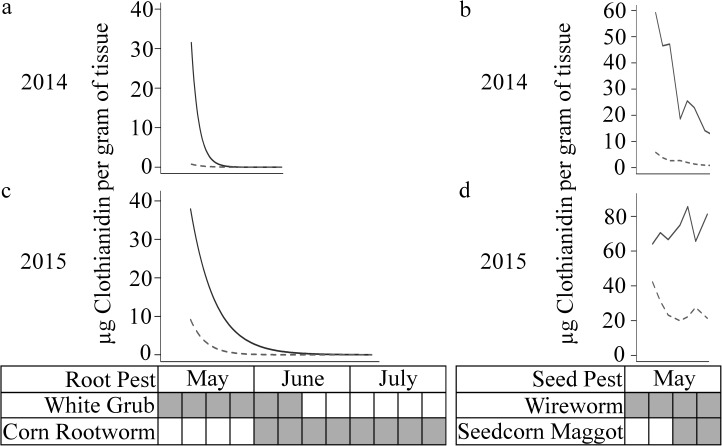
Values of μg clothianidin per g of plant tissue fit to a first order decay equation with time as a predictor. The root region is represented by graphs a and c whereas the seed region by graphs b and d. Actual concentrations for the seed region are displayed given the poor fit of predicted values. Dashed and solid lines represent the 0.25 and 1.25 mg /clothianidin application rates. The pest activity period is displayed underneath the graphs with activity indicated by a filled in box.

The first western corn rootworm neonate larvae of the season were observed at 27 and 28 DPP in 2014 and 2015, respectively (Fig 4). Larval emergence from overwintered eggs typically occurs during late May to early June in this region of Indiana [56]. Our seedcorn maggot model predicted egg hatch at planting and one day before in 2014 and 2015, with larval development (the damaging stage) being completed at 16 DPP and 12 DPP for 2014 and 2015, respectively.

No defined “attack period” for our study region is reported in peer-reviewed literature for white grubs and wireworm beyond that of a generalized early season root and seed feeding pest [17], however extension literature [50] places white grub and wireworm attack from mid-April to mid and late June respectively in Indiana; these estimates are necessarily variable as they depend largely upon degree day accumulations. For the purpose of this project, the end of white grub and wireworm attack was considered June 15^th^ (41 DPP) and June 30^th^ (56 DPP) respectively.

### Determination of protection window

**Fig 5:**
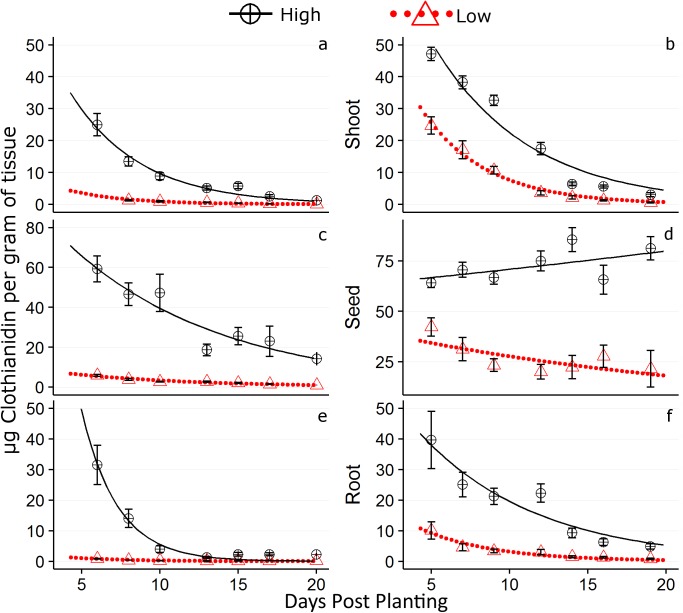
Values of μg clothianidin per g of plant tissue with standard error bars. The 2014 data are represented by graphs (a), (c), and (e), and 2015 data by graphs (b), (d), and (f). The first 20 days post planting (DPP) are shown. Concentrations as predicted by the first order exponential decay equation are represented by a dotted red line and solid black line for the respective 0.25 and 1.25 mg /clothianidin application rates.

The exponential decay equation explained 34.4–46.1% and 54.1–86% of the variance in tissue-bound AI concentration for the respective root and shoot regions of treated plants. In comparison to NST plants in both years, “Naked” plants had lower R^2^ values indicating a smaller proportion of explained variance resultant from the DPP predictor and exponential fit (Table 2). Root and shoot regions had larger R^2^ values than their corresponding seed region indicating tissue-bound AI concentration better conformed to the exponential decay prediction. This was characterized in the root region by a flattening out of the decay curve around 17 and 20 DPP for the “Low” treatment and 15 and 25 DPP for the “High” treatment in 2014 and 2015 respectively (Figs 4 and 5). By taking the average of both years for each treatment type, the protection window in the root region for “Low” and “High” treatments was considered to be 18.5 DPP and 20 DPP respectively. For the shoot region, the flattening out of the decay curve occurred around 17 and 22 DPP for the “Low” treatment and 20 and 33 DPP for the “High” treatment in 2014 and 2015 respectively. The decay curve was not used to estimate a protection window for the seed region given its overall poor fit to the data.

**Table 2:** R^2^ values describing the fit of the translocation data as a function of days post planting (DPP) for clothianidin as estimated by the exponential decay function: *C* = *C*_0_*e*^−*kt*^.

Region	Treatment	2014	2015
Root	Naked	0.101	0.304
	Fung	0.499	0.618
	Low	0.461	0.344
	High	0.421	0.437
Seed	Naked	0.272	0.214
	Fung	0.287	0.400
	Low	0.494	0.041
	High	0.244	0.027
Shoot	Naked	0.351	0.344
	Fung	0.523	0.681
	Low	0.585	0.731
	High	0.541	0.86

A significant multivariate treatment*sampling date interaction was recorded once in the Root 2014 model (Table 3) indicating relative treatment differences of in-plant CLO concentrations were similar across the sampling period in the remaining models. As the univariate treatment effects in all models remained highly significant (P<0.001) until 20 DPP (Table 4), only contrasts taking place on or after 15 DPP were considered. Visual inspection of decay curves confirmed this initial assessment and further reduced the number of *a priori* contrasts made.

**Table 3:** F-values and estimated degrees of freedom (df) for the multivariate repeated-measures ANOVA model describing in-plant concentrations of clothianidin over the sampling period in 2014 and 2015 for the three plant regions (Root, Seed, Shoot).

Region	2014 Factor	df	F-value	2015 Factor	df	F-value
Root	Time	7,3	418.22^***^	Time	8,1	385.83*
	Treatment*Time	21,9.17	4.64*	Treatment*Time	24,3.50	4.26
Seed	Time	6,4	314.37^***^	Time	6,3	17.07*
	Treatment*Time	18,11.80	1.15	Treatment*Time	18,8.97	1.93
Shoot	Time	7,3	181.26^***^	Time	8,1	328.45*
	Treatment*Time	21,9.17	2.27	Treatment*Time	24,3.50	1.25

*P < 0.05.

**P < 0.01.

***P < 0.001.

**Table 4:** Univariate F-values and degrees of freedom (df) generated following the multivariate repeated-measures ANOVA model describing influence of initial clothianidin seed treatment (Treat) on in-plant concentrations of clothianidin over the course of multiple days post planting (DPP) in 2014 and 2015 and three plant regions (Root, Seed, Shoot).

2014			F-value								
Region	Factor	df	6 DPP	8 DPP	10 DPP	13 DPP	15 DPP	17 DPP	20 DPP	34 DPP	
Root	Treat	3,9	142.15^***^	79.27^***^	32.59^***^	67.51^***^	177.66^***^	21.51^***^	17.20^***^	3.69	
Seed	Treat	3,9	71.67^***^	112^***^	63.74^***^	84.58^***^	143.84^***^	52.19^***^	24.07^***^		
Shoot	Treat	3,9	40.78^***^	121.11^***^	116.15^***^	64.44^***^	108.55^***^	91.78^***^	37.15^***^	1.65	
2015											
Region	Factor	df	5 DPP	7 DPP	9 DPP	12 DPP	14 DPP	16 DPP	19 DPP	47 DPP	61 DPP
Root	Treat	3,8	52.89^***^	24.97^***^	321.89^***^	50.81^***^	159.45^***^	71.75^***^	55.24^***^	2.34	2.77
Seed	Treat	3,8	209.29^***^	429.13^***^	1356.72^***^	111.42^***^	86.15^***^	133.09^***^	35.03^***^		
Shoot	Treat	3,8	429.43^***^	79.38^***^	229.14^***^	37.17^***^	101.01^***^	93.13^***^	85.25^***^	1.87	0.19

*P < 0.05.

**P < 0.01.

***P < 0.001.

In 2014, concentrations were similar or converged (*P* > 0.05) at 17, 20, and 34 DPP for “Naked”+“Fungicide” vs “Low” contrasts for the respective root, seed, and shoot tissues, but not for the “Naked”+“Fungicide” vs “High” contrasts which remained different throughout the sampling period (Table 5), with the exception of the shoot region at 34 DPP. In 2015, this did not occur for the root or shoot region until 47 DPP for either contrast set. It is possible that concentrations converged at an earlier period however freezer malfunction resulted in the loss of 33 DPP samples.

**Table 5:** F-values of a priori contrasts comparing untreated maize seed (Fungicide + Naked) to 0.25 mg clothianidin/kernel (Low) and 1.25 mg clothianidin/kernel (High) at various days post planting (DPP) for three different plant regions (Root, Seed, Shoot) in 2014 and 2015.

		2014		2015	
Region	Contrast	DPP	F-value	DPP	F-value
Root	Fungicide+Naked vs Low	15	F_1,9_ = 38.86^***^	19	F_1,8_ = 49.46^***^
		17	F_1,9_ = 1.89	47	F_1,8_ = 5.11
		20	F_1,9_ = 0.66	61	F_1,8_ = 2.81
	Fungicide+Naked vs High	17	F_1,9_ = 57.59^***^	19	F_1,8_ = 162.78^***^
		20	F_1,9_ = 47.08^***^	47	F_1,8_ = 4.09
		34	F_1,9_ = 10.13^*^	61	F_1,8_ = 1.41
Seed	Fungicide+Naked vs Low	17	F_1,9_ = 18.98^**^	19	F_1,8_ = 44.07^***^
		20	F_1,9_ = 3.97		
	Fungicide+Naked vs High	17	F_1,9_ = 155.55^***^	19	F_1,8_ = 87.40^***^
		20	F_1,9_ = 69.20^***^		
Shoot	Fungicide+Naked vs Low	17	F_1,9_ = 26.69^***^	19	F_1,8_ = 84.97^***^
		20	F_1,9_ = 8.17^*^	47	F_1,8_ = 0.38
		34	F_1,9_ = 0.56		
	Fungicide+Naked vs High	20	F_1,9_ = 107.03^***^	19	F_1,8_ = 237.63^***^
		34	F_1,9_ = 4.47	47	F_1,8_ = 4.77

*P < 0.05.

**P < 0.01.

***P < 0.001.

To compare pest phenology to concentration data, the shoot region was considered protected until 34 DPP regardless of CLO application rate and treated seed was considered protected for the entirety of the seed recovery period (up to 20 DPP in both years) despite concentration convergence at 17 DPP in the “Naked”+“Fungicide” vs “Low” contrasts (Table 5). The root region was considered protected up to 34 and 47 DPP for the respective “Low” and “High” treatment rates. Protection was considered to last up to 34 DPP for the “Low” treatment rate as a compromise between 2014 and 2015 data. Root protection was first lost at 17 DPP in 2014 but was still provided at 19 DPP in 2015. As a freezer malfunction resulted in the loss of 33 DPP samples in 2015, the next possible sampling date was 47 DPP in which protection had already been lost. It is unknown whether protection had been lost by 33 DPP in 2015, however by selecting 34 DPP as the date of protection loss, we balance the possibility of underestimating the protection window based on 2014 data and overestimating the protection window based on 2015 data.

## Discussion

These results are the first to use field experiments to quantify in-plant concentrations of CLO, the principal neonicotinoid AI currently used in North American maize production, and demonstrate a rapid reduction in tissue-bound CLO beginning in the days following seed sowing. An exponential decay equation [53] provides explanatory power in describing the relationship between CLO concentrations solely as a function of time in the root and shoot region of treated plants. Given the water solubility of CLO (0.327 g/L at 20°C [16]), and the low percent of AI remaining on the seed at the first sampling date, it is likely the removal of CLO from the seed surface was rapid following seed sowing and followed an exponential decay pattern; our sampling protocol may have been initiated too late (5 and 6 DPP in 2015 and 2014 respectively) to observe this trend fully. This may explain why the seed region in all treatments had the lowest R^2^ values associated with the exponential decay equation (Table 2). Furthermore, the combination of CLO’s high water solubility and long soil half-life (277–1386 days [27]) is likely underlying our observations of CLO in untreated plant tissues, either as a result of lateral movement between plots and/or as a residue from the previous planting season. This is not unexpected as expression of neonicotinoids in non-target plants has previously been attributed to subsurface movement [38,39].

These data also provide a potential mechanism to explain a range of field observations from previously published literature. Numerous studies have reported inconsistent yield benefits of NSTs in maize, including finding no advantages of the NST approach when compared to maize seed having no insecticide applied [57–59]. Similarly, our study found no statistical differences in stand count, root ratings, or yield between treated and untreated seed in both years. While the presence of root feeding pests was documented in both years as evidenced by root ratings (Table 1), economic injury was only observed in the 2015 “Low” and “Fungicide” treatments and only if NST cost was assumed to be <$27/ha and <$32.5/ha respectively. In other words, no economic benefit would be realized if the respective application costs were >$27/ha and >$32/ha for the “Low” and “Fungicide” treatments. This interaction between insect damage, crop yield, and insecticide cost is expressed in the following equation [60]:

$$EIL=\frac{C}{V*b*K}$$

Where the economic injury level (*EIL*) is defined as management cost per area (*C*) over market value per produce unit (*V*) multiplied by yield loss per insect (*b*) and the proportionate reduction in potential injury (*K*).

Out of necessity, we assumed a range of insect control costs ($17.5-$55/ha) for *C* because the actual cost is not disclosed to growers or available in the published literature. Estimates range widely from between ~$17.5/ha [61,62] to $37.5/ha [63]). Below, we outline two sets of calculations that shed light on the potential fit for this pest management approach.

Using a value of *K* of 1 (i.e. 100% reduction in pest injury) is admittedly unrealistic and unattainable, but provides a best case option for exploring different scenarios given market values and insect control costs influence EIL calculation. For example, transposing our observed 2015 damage results and using the recent 5-year high for Indiana maize market values of $28.46/100 kg in 2012 [47], we would expect economic injury in the 2015 “Naked”, “Low” and “Fungicide” treatments with respective control costs <$26, <$51, and <$62/ha (Table 1 and Fig 2). However a more informative approach for growers is to use the 2014–15 average ($14.96/100 kg), which coincides with the 2016 mean market values ($14.87/100 kg [64]) to calculate the EIL; values are approximately 50% lower than 2012 values resulting in control costs of $27/ha and $32.5/ha for respective “Low” and “Fungicide” treatments. While the 2012 example demonstrates spending an initial ~$17.50/ha on NST may be justified under high market prices or high pest pressure, lower market prices (2014–16) limit any additional control tactics a grower can afford.

The results of previous reports [57–59] and the findings we report here suggest that *K* is lower than 1. Comparison of damage in the untreated versus treated plants allows us to estimate *K*. In 2015, the average root rating for the “Naked” and “Fungicide” treatments was 0.3245 and the “High” was 0.128 leading to an estimated *K* value of 0.395. Using this value, none of the tested treatments reached economic injury as the lowest tested treatment cost ($17.50/ha) would require a minimum root rating of 0.53 to reach economic injury levels. A less efficacious pest management approach effectively raises the economic threshold.

Our economic threshold estimates using these data provide a starting point for discussion, but the overall benefit of NSTs cannot be fully assessed without knowledge of the actual cost of NSTs for maize growers. Maize seed without NSTs is increasingly scarce in the current marketplace [5]. A true “free market” approach to seed availability, including a wide selection of readily available NST-free maize seed would provide a basis for cost comparison while allowing producers, consultants and researchers to readily make on-farm comparisons and determine if and when NST costs are justified.

In assessing potential benefits of this approach, we chose to use in-plant CLO concentrations to construct a pest management window for maize plants grown from treated seeds. Planned contrasts provided a highly conservative estimate of the date after planting at which the CLO concentration in treated plant tissue was statistically similar to that of untreated plant tissue. This approach to the development of a pest management window is highly conservative, in that it assumes that even very low levels of the AI in plant tissue is likely to provide pest management benefit, a notion that has not been tested empirically in the lab or field. This is the most parsimonious initial approach for interpreting these data from a pest management standpoint because oral LD_50_ concentration data for NSTs have not been reported for the target pest insects.

This study also demonstrates that when deploying NSTs, consideration should be given to pest biology, more specifically to how the pest’s activity window and region of attack overlap periods where a pest management benefit of NSTs could be expected. In the case of the key pest of maize, western corn rootworm, the damaging larval stage was active starting at 27 and 28 DPP in 2014 and 2015 respectively (Fig 4). As *a priori* contrasts suggested CLO STs at the “High” rootworm rate [11,12] stopped providing protection to the root tissue by 47 DPP, ~20 days of western corn rootworm protection were provided (Table 5). This may be a sufficient window and insecticide concentration level (from 31.47 μg/g (6 DPP) to 0.02 μg/g (47 DPP) for “High” root tissue in 2015) to deter or kill neonate western corn rootworm larvae, although no data exist to support or refute this hypothesis. Alternatively, decay curve analysis shows the likely root protection window extended to 18.5 and 20 DPP for the respective “Low” and “High” treatments, ending well before western corn rootworm emergence and presenting a poor fit with the phenology of this key pest. While it is possible that the ambient AI concentration in soil around roots is high enough to provide control, this hypothesis has not been tested experimentally.

A similar result was obtained with white grubs, in which *a priori* contrasts predicted root protection for all of the white grub active period at the “High” rate and all but 7 days at the “Low” rate. However exponential decay analysis estimated root tissue as unprotected for 22.5 and 21 days for “Low” and “High” rates, respectively. The CLO STs failed to provide protection from western corn rootworm and white grub for the entirety of the pest activity window, but this is not the case for seedcorn maggot and wireworms where both *a priori* contrasts suggested CLO STs provided some protection to the seed region for the vast majority of the seed recovery period (up to 20 DPP). Our data suggest that fields with a history of wireworm, seedcorn maggot, or white grub, may benefit from the use of NSTs as a seed protectant during the first 20 DPP as their activity window coincides with the window of highest AI concentration within plant tissues. The root region protection window is less clear as conservative estimates indicate 20–21 days of western corn rootworm protection at the “High” rootworm rate and at least 34–47 days of white grub protection at the “Low” and “High” rates.

Finally, the rapid decrease in concentration of insecticide within plant tissues points to a broader question for current NST approaches. The NSTs are marketed as a targeted pesticide delivery system [1], however our findings demonstrate that NSTs may be a highly inefficient approach to applying active ingredients to plant tissues where insects will ingest them. In sum, less than 1.5% of the initial seed-applied AI was recovered in whole plant translocations. When looking at the root tissue alone, a maximum of 0.262% of the initial amount was translocated (Fig 3), although it is unknown whether these levels are sufficient for pest management. For reference, a higher percentage of the initial ST amount applied to seeds has been reported as lost during planting due to abrasion ((0.437%) [65]). The shoot region however translocated a maximum of 1.18%, a likely result of the high xylem mobility many of the neonicotinoids possess (Bonmatin et al. 2015). Similar translocation efficacy, under field conditions is expected for thiamethoxam, the second most widely-used NST in maize, which has a water solubility value approximately 10-fold higher than that of CLO [20, 66]. These results also contrast with those of Sur and Stork [23] whose study reported 20% translocation of imidacloprid STs in maize. The authors mention that their translocation values may be inflated as the study was conducted in a greenhouse, reducing impacts of UV photolysis and weather conditions, and that the amount of soil in the plant boxes compared to the root mass exaggerated imidacloprid uptake. This is likely a key limitation of that study and may explain why our recovery from a field experiment was much lower; AI that would be lost to the water table in the field could remain in greenhouse enclosures and be available for uptake by plants throughout the season in this relatively closed system.

Determining the environmental fate of the remaining ca. 98% of active ingredient used in NST is an area primed for further research. The intrinsic characteristics of CLO, thiamethoxam and imidacloprid, and a growing body of literature reporting neonicotinoids in water lends support to the interpretation that the remainder of the ST is rapidly lost to the environment in ground and surface water [31–33, 35, 36]. The high water solubilities of the compounds most commonly used in NST applications make it unlikely that they will reside near the target plant’s relatively limited rhizosphere long enough to be absorbed by the plant once they are not on the seed. This may explain why samples in 2015 generally had a higher % AI translocated (Fig 4) as cumulative rainfall in 2015 was 2.66-fold less than over the same 20 DPP period in 2014. This may also explain why the concentrations reported in our study depart from those of Myresiotis et al. [22]. While plants were regularly irrigated in the Myresiotis et al. [22] study, the authors mention they made sure the entire volume of water remained within the rhizosphere soil in an effort to avoid leaching. Using seed treated with 0.1 mg thiamethoxam per kernel, Myresiotis et al. [22] found shoot concentrations of 0.62 and 0.13 μg thiamethoxam/g at 21 and 36 DPP respectively. Using the “Low” rate of 0.25 mg CLO per kernel, our study found in plant concentrations of 0.086 and 0.007 μg CLO/g at 20 and 34 DPP respectively in 2014. Despite the higher initial application rate of our seed, the Myresiotis et al. [22] study had overall larger AI concentrations in shoot tissue which is likely due to differences in experimental design. AI that was lost to leaching in our field study would have been preserved in a potted plant study with constrained irrigation.

## Conclusions

While the highest in-plant neonicotinoid concentrations reported from this research may provide some control of early season root and seed pests, the relatively brief window of high AI concentrations poorly coincides with the phenology of the key maize pest in the USA. This coupled with the sporadic occurrence of economic infestations [17] of secondary pests indicates that most US maize producers are unlikely to realize benefits from the NST approach. Furthermore, the widespread prophylactic application of NSTs [5] and their high water solubility, coupled with the limited translocation efficiency reported in this study, provide a mechanism to explain increasing detections of NST compounds in non-target lands and waterways [31–33, 35,36].

## Supporting information

S1 Dataset 2014 and 2015 clothianidin concentration data. (XLSX)

S2 Dataset 2014 and 2015 root rating data. (XLSX)

S3 Dataset 2014 and 2015 stand count data. (XLSX)

S4 Dataset 2014 and 2015 yield data. (XLSX)

S1 Text Modified QuECHeRs protocol for homogenization and machine settings. (DOCX)
