# Correction: Translocation of the neonicotinoid seed treatment clothianidin in maize

**DOI:** 10.1371/journal.pone.0186527

**Published:** 2017-10-11

**Authors:** Adam Alford, Christian H. Krupke

One data point is inadvertently omitted from the upper left graph in Fig 5. The authors have provided the corrected version here.

**Fig 5:**
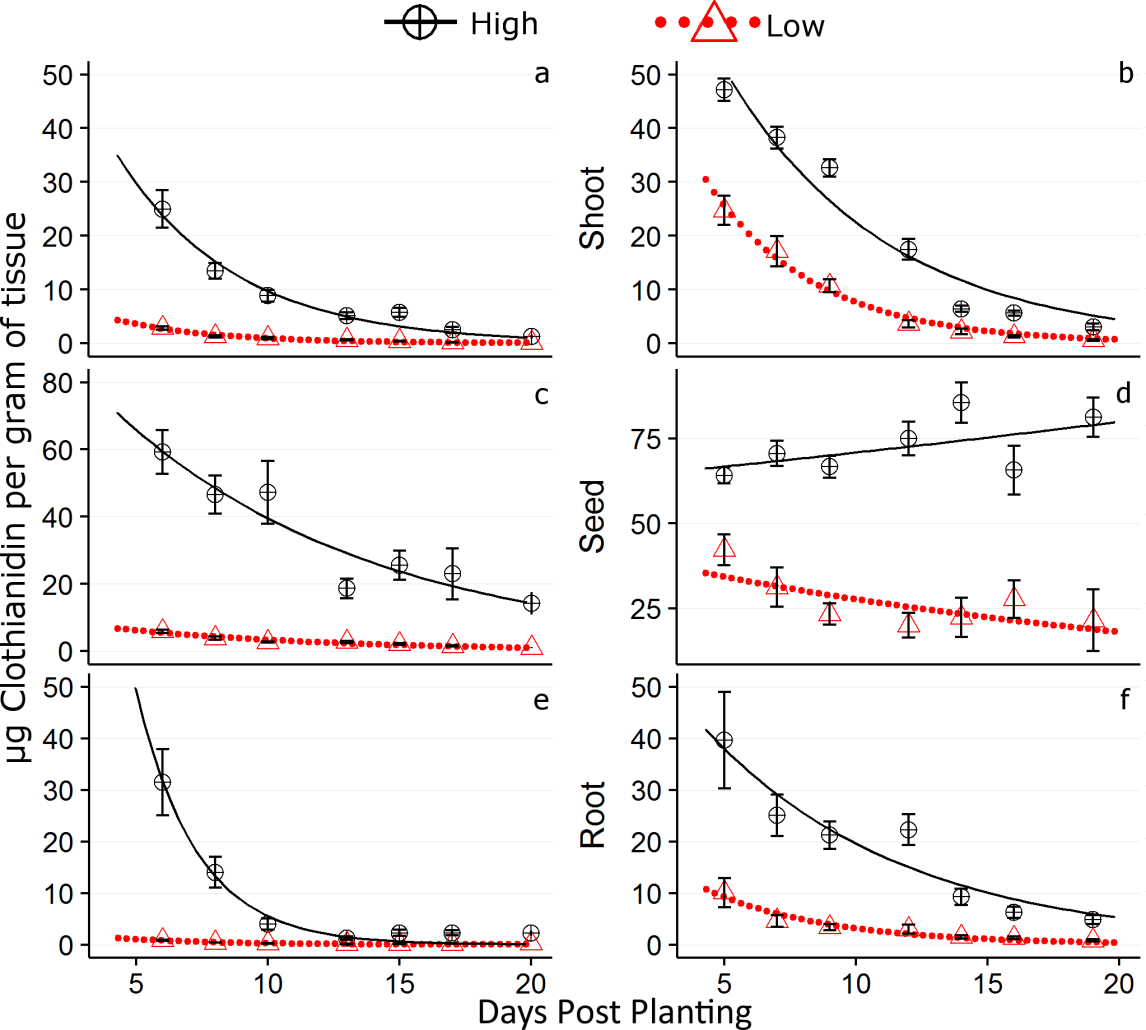
Values of μg clothianidin per g of plant tissue with standard error bars. The 2014 data are represented by graphs (a), (c), and (e), and 2015 data by graphs (b), (d), and (f). The first 20 days post planting (DPP) are shown. Concentrations as predicted by the first order exponential decay equation are represented by a dotted red line and solid black line for the respective 0.25 and 1.25 mg /clothianidin application rates.

Several data points were inadvertently omitted from the data set used for analysis (S1 Dataset). These data points should be included, and Tables 2, 3, 4 and 5 should be changed due to this addition. This does not affect the conclusions of the paper.

**Table 2:** R^2^ values describing the fit of the translocation data as a function of days post planting (DPP) for clothianidin as estimated by the exponential decay function: *C* = *C*_0_*e*^−*kt*^.

Region	Treatment	2014	2015
Root	Naked	0.101	0.306
	Fung	0.499	0.618
	Low	0.461	0.344
	High	0.421	0.437
Seed	Naked	0.272	0.210
	Fung	0.287	0.400
	Low	0.494	0.041
	High	0.244	0.027
Shoot	Naked	0.351	0.347
	Fung	0.523	0.681
	Low	0.585	0.731
	High	0.541	0.86

*P < 0.05 **P < 0.01 ***P < 0.001

**Table 3:** F-values and estimated degrees of freedom (df) for the multivariate repeated-measures ANOVA model describing in-plant concentrations of clothianidin over the sampling period in 2014 and 2015 for the three plant regions (Root, Seed, Shoot).

Region	2014 Factor	df	F-value	2015 Factor	df	F-value
Root	Time	7,3	418.22^***^	Time	8,1	391.84^*^
	Treatment*Time	21,9.17	4.64^*^	Treatment*Time	24,3.50	4.43
Seed	Time	6,4	314.37^***^	Time	6,3	14.05^*^
	Treatment*Time	18,11.80	1.15	Treatment*Time	18,8.97	2.42
Shoot	Time	7,3	181.26^***^	Time	8,1	328.26^*^
	Treatment*Time	21,9.17	2.27	Treatment*Time	24,3.50	1.25

*P < 0.05

**P < 0.01

***P < 0.001

**Table 4:** Univariate F-values and degrees of freedom (df) generated following the multivariate repeated-measures ANOVA model describing influence of initial clothianidin seed treatment (Treat) on in-plant concentrations of clothianidin over the course of multiple days post planting (DPP) in 2014 and 2015 and three plant regions (Root, Seed, Shoot).

2014			F-value								
Region	Factor	df	6 DPP	8 DPP	10 DPP	13 DPP	15 DPP	17 DPP	20 DPP	34 DPP	
Root	Treat	3,9	142.15^***^	79.27^***^	32.59^***^	67.51^***^	177.66^***^	21.51^***^	17.20^***^	3.69	
Seed	Treat	3,9	71.67^***^	112^***^	63.74^***^	84.58^***^	143.84^***^	52.19^***^	24.07^***^		
Shoot	Treat	3,9	40.78^***^	121.11^***^	116.15^***^	64.44^***^	108.55^***^	91.78^***^	37.15^***^	1.65	
2015											
Region	Factor	df	5 DPP	7 DPP	9 DPP	12 DPP	14 DPP	16 DPP	19 DPP	47 DPP	61 DPP
Root	Treat	3,8	52.89^***^	24.97^***^	321.89^***^	50.81^***^	159.45^***^	71.75^***^	62.14^***^	2.34	2.77
Seed	Treat	3,8	424.34^***^	369.71^***^	715.73^***^	128.60^***^	82.35^***^	93.66^***^	44.11^***^		
Shoot	Treat	3,8	429.43^***^	79.38^***^	229.14^***^	37.17^***^	101.01^***^	93.13^***^	87.86^***^	1.87	0.19

*P < 0.05

**P < 0.01

***P < 0.001

**Table 5:** F-values of a priori contrasts comparing untreated maize seed (Fungicide + Naked) to 0.25 mg clothianidin/kernel (Low) and 1.25 mg clothianidin/kernel (High) at various days post planting (DPP) for three different plant regions (Root, Seed, Shoot) in 2014 and 2015.

		2014		2015	
Region	Contrast	DPP	F-value	DPP	F-value
Root	Fungicide+Naked vs Low	15	F_1,9_ = 38.86^***^	19	F_1,8_ = 57.11^***^
		17	F_1,9_ = 1.89	47	F_1,8_ = 5.11
		20	F_1,9_ = 0.66	61	F_1,8_ = 2.81
	Fungicide+Naked vs High	17	F_1,9_ = 57.59^***^	19	F_1,8_ = 180.27^***^
		20	F_1,9_ = 47.08^***^	47	F_1,8_ = 4.09
		34	F_1,9_ = 10.13^*^	61	F_1,8_ = 1.41
Seed	Fungicide+Naked vs Low	17	F_1,9_ = 18.98^**^	19	F_1,8_ = 53.89^***^
		20	F_1,9_ = 3.97		
	Fungicide+Naked vs High	17	F_1,9_ = 155.55^***^	19	F_1,8_ = 110.31^***^
		20	F_1,9_ = 69.20^***^		
Shoot	Fungicide+Naked vs Low	17	F_1,9_ = 26.69^***^	19	F_1,8_ = 89.05^***^
		20	F_1,9_ = 8.17^*^	47	F_1,8_ = 0.38
		34	F_1,9_ = 0.56		
	Fungicide+Naked vs High	20	F_1,9_ = 107.03^***^	19	F_1,8_ = 242.76^***^
		34	F_1,9_ = 4.47	47	F_1,8_ = 4.77

*P < 0.05

**P < 0.01

***P < 0.001

Please see the complete, correct S1 Dataset below.

## Supporting information

S1 Dataset 2014 and 2015 clothianidin concentration data. (XLSX)
