# Supplementary material for: Translocation of the neonicotinoid seed treatment clothianidin in maize
Source: PLoS One. 2017 Mar 10;12(3):e0173836. doi: 10.1371/journal.pone.0173836 (PMC5345846; doi:10.1371/journal.pone.0173836)
Supplement: S1 Text — (DOCX) [file pone.0173836.s005.docx]

Supporting Information for

**Translocation of the neonicotinoid seed treatment clothianidin in maize**

Adam M. Alford^1*^, Christian H. Krupke^1^

^1^Department of Entomology, Purdue University, West Lafayette, Indiana, United States

* Corresponding author

E-mail: AdamMAlford@gmail.com

A Precellys^®^ 24-Dual homogenizer along with Precellys^®^ 7 mL homogenization tubes (Bertin Technologies, France) were used to conduct all homogenizations. Each region (root, seed, shoot) of a given sample was homogenized in 4 mL of HPLC grade acetonitrile (ACN) solvent. The homogenate was then transferred to a Falcon^TM^ 15 mL conical centrifuge tube and the empty homogenization tube was washed with an additional 4 mL of ACN. The ACN wash was then added to the homogenate followed by an additional 2 mL of ACN to take the total ACN volume up to 10 mL. Cleaning salts (0.5 g Sodium chloride, 2 g magnesium sulfate, 0.25 g sodium hydrogencitrate sesquihydrate) and 10 µL of a 10 ng/ µL d3-clothianidin (Sigma-Aldrich, St. Louis, MO, USA) internal standard were added to the samples which were then centrifuged at 4˚C for 10 min at 2500 rpm. A 1 mL portion of the ACN supernatant was further cleaned in 2 mL QuEChERS dispersive Solid Phase Extraction (dSPE) tubes for use with fruits and vegetables (Agilent Technologies, Santa Clara CA, USA). The dSPE tubes were vortexed for 10 min and centrifuged at 15,000 rpm for 5 min. The ACN supernatant was then transferred to a 1.5 mL Eppendorf tube and concentrated in a SpeedVac until pellet formation, after which the pellet was re-suspended in 100 µL ACN and analyzed with an Agilent 6460 Triple Quadrupole (QQQ) (Santa Clara, CA, USA) using Liquid Chromatography tandem Mass Spectrometry (LC-MS). Clothianidin quantification (Limit of Detection (LOD) 0.1 ng/g) was achieved by comparing the ratio of deuterium labelled-clothianidin (d3-clothianidin) to that extracted from the plant tissue.

An Agilent 1200 Rapid Resolution liquid chromatography (LC) system coupled to an Agilent 6460 series QQQ mass spectrometer (MS) was used to analyze pesticides in each sample. An Agilent Zorbax SB-Phenyl 4.6 x 150 mm, 5 µm column was used for LC separation (Agilent Technologies, Santa Clara, CA). The buffers were (A) water + 5 mM ammonium acetate + 0.1 % formic acid and (B) acetonitrile (90%) + 5 mM ammonium acetate (10%) + 0.1% formic acid. The linear LC gradient was as follows: time 0 min, 5 % B; time 0.5 min, 5 % B; time 8 min, 100 % B; time 10 min, 100 % B; time 11 min, 5 % B; time 15 min, 5 % B. The flow rate was 0.8 mL/min. Multiple reaction monitoring was used for MS analysis. The data were acquired in positive electrospray ionization (ESI) mode. Precursor ions of d3-clothianidin and clothianidin had respective molecular weights of 253 and 249.9 g/mol with product ions of 172.1 and 169 g/mol. For both ions, the dwell was set at 50 msec, fragmentor voltage at 70, collision energy at 10V, cell accelerator voltage at 1, with positive polarity. The jet stream ESI interface had a gas temperature of 330°C, gas flow rate of 10 L/min, nebulizer pressure of 35 psi, sheath gas temperature of 250°C, sheath gas flow rate of 7 L/min, capillary voltage of 4000 V in positive mode, and nozzle voltage of 1000 V. The ΔEMV voltage was 300. All data were analyzed with Agilent Masshunter Quantitative Analysis (Version B.06.00).
